# Supplementary material for: Mechanisms governing the pioneering and redistribution capabilities of the non-classical pioneer PU.1
Source: Nat Commun. 2020 Jan 21;11:402. doi: 10.1038/s41467-019-13960-2 (PMC6972792; doi:10.1038/s41467-019-13960-2)
Supplement: Supplementary file 7 — Source data [file 41467_2019_13960_MOESM7_ESM.zip › Source_Data/Figure6/Figure6B_MotifScanOutput/ETS1_mutPU.1/homerResults/motif7.similar.html]

motif7

## Information for motif7

A
C
G
T
C
G
A
T
T
G
C
A
A
G
T
C
A
C
T
G
A
C
G
T
G
A
T
C
  
Reverse Opposite:  

C
T
A
G
T
G
C
A
A
G
T
C
A
C
T
G
A
C
G
T
C
G
T
A
C
G
T
A
  

|  |  |
| --- | --- |
| p-value: | 1e-205 |
| log p-value: | -4.733e+02 |
| Information Content per bp: | 1.737 |
| Number of Target Sequences with motif | 1945.0 |
| Percentage of Target Sequences with motif | 14.42% |
| Number of Background Sequences with motif | 1949.2 |
| Percentage of Background Sequences with motif | 5.56% |
| Average Position of motif in Targets | 101.2 +/- 56.7bp |
| Average Position of motif in Background | 97.0 +/- 68.8bp |
| Strand Bias (log2 ratio + to - strand density) | -0.2 |
| Multiplicity (# of sites on avg that occur together) | 1.05 |
| Motif File: | file (matrix) reverse opposite |

### Similar de novo motifs found

|  |  |  |  |  |  |  |  |
| --- | --- | --- | --- | --- | --- | --- | --- |
| Rank | Match Score | Redundant Motif | P-value | log P-value | % of Targets | % of Background | Motif file |
| 1 | 0.835 | A G T C A C T G A T G C C G T A C G T A C A G T T G C A | 1e-205 | -473.022975 | 42.87% | 28.17% | motif file (matrix) |
| 2 | 0.868 | C G A T C A G T T C G A A G T C A C T G G C A T G T A C T C G A C A G T | 1e-30 | -69.259284 | 4.35% | 2.32% | motif file (matrix) |
| 3 | 0.832 | A T C G A C G T A C G T A C G T C T G A A G T C A C T G C T A G A G T C | 1e-19 | -44.476896 | 1.25% | 0.45% | motif file (matrix) |
